# Sensorimotor Incongruence and Body Perception: An Experimental Investigation

**DOI:** 10.3389/fnhum.2013.00310

**Published:** 2013-06-24

**Authors:** Jens Foell, Robin Bekrater-Bodmann, Candida S. McCabe, Herta Flor

**Affiliations:** ^1^Department of Cognitive and Clinical Neuroscience, Central Institute of Mental Health, Medical Faculty Mannheim, Heidelberg University, Mannheim, Germany; ^2^Department of Psychology, Florida State University, Tallahassee, FL, USA; ^3^University of the West of England, Bristol, UK; ^4^Royal National Hospital for Rheumatic Diseases, Bath, UK

**Keywords:** pain, somatosensory system, sensory-motor incongruence, illusion, body representation, mirror

## Abstract

**Objectives:** Several studies have shown that mirrored arm or leg movements can induce altered body sensations. This includes the alleviation of chronic pain using congruent mirror feedback and the induction of abnormal sensation in healthy participants using incongruent mirror feedback. Prior research has identified neuronal and conceptual mechanisms of these phenomena. With the rising application of behavior-based methods for pain relief, a structured investigation of these reported effects seems necessary.

**Methods:** We investigated a mirror setup that included congruent and incongruent hand and arm movements in 113 healthy participants and assessed the occurrence and intensity of unusual physical experiences such as pain, the sensation of missing or additional limbs, or changes in weight or temperature. A wooden surface instead of a mirror condition served as control.

**Results:** As reported earlier, mirrored movements led to a variety of subjective reactions in both the congruent and incongruent movement condition, with the sensation of possessing a third limb being significantly more intense and frequent in the incongruent mirror condition. Reports of illusory pain were not more frequent during mirrored than during non-mirrored movements.

**Conclusion:** These results suggest that, while all mirrored hand movements induce abnormal body perceptions, the experience of an extra limb is most pronounced in the incongruent mirror movement condition. The frequent sensation of having a third arm may be related to brain processes designed to integrate input from several senses in a meaningful manner. Painful sensations are not more frequent or intense when a mirror is present.

## Introduction

In order to produce and control complex and precise body movements, the brain constantly processes and integrates input from several senses, such as the visual and sensorimotor domains. This is a rather complex task which can be disturbed by deliberately giving contradictory information to two or more senses. The reactions to such disturbances are diverse and might have important clinical implications. The critical interaction between motor movements and the central nervous system is based on von Holst and Mittelstaedt (1950), who postulated that every motor command (i.e., efference) is processed using a specific expectation of its effect (i.e., the efference copy). Incongruent feedback between the motor system and vision constitutes a mismatch between the expected and factual response of the motor action. On a neural level, such a conflict between the senses seems to be monitored by the dorsolateral and ventral prefrontal cortices (Fink et al., 1999), both of which have been shown to also modulate pain processing in humans (Ploghaus et al., 1999; Lorenz et al., 2003). Consequently, Harris (1999) proposed a model which states that such discordance between motor intent and its sensory feedback is able to elicit pain as a warning mechanism. If correct, this model may explain certain pain phenomena that occur in the absence of physical painful stimuli, such as phantom limb pain (PLP).

McCabe et al. (2005) examined the potential of mirrored movements and obscured visual feedback to cause unusual sensations, including pain. In their paradigm, one limb was hidden by either a non-reflective whiteboard or mirror. In the latter scenario, the reflection of the observed limb seemingly replaced the hidden limb. This setup enabled observation of the impact on an individual of a graded manipulation of conflict between proprioception, vision, and motor intention. The four intervention stages included two movement conditions without visual feedback (viewing a whiteboard with congruent and incongruent movements); accurate visual feedback of the moving limb but with minimal distortion/distancing via a mirror (congruent movements whilst viewing the mirror); and finally, incorrect visual feedback of the moving limb (incongruent movement whilst viewing the mirror).

Imaging studies using magnetoencephalography have shown that visual information plays a key role in activation of somatosensory areas during movement related tasks with increased activation of the secondary somatosensory cortex and parietal cortex when unexpected visual feedback is received (Wasaka and Kakigi, 2012). Subjects’ “vulnerability” to a sensorimotor mismatch was determined by the sum of the number of conditions which generated novel sensory perceptions, that is the most “vulnerable” reported new sensations in all four intervention conditions. Using this paradigm, McCabe et al. (2005) reported that 66% of participants described a new sensory response at some stage in the protocol with the highest incidence of report in the incongruent mirror condition (59%). This pattern of response was also seen for pain reports with slight pain (<2/10 on a visual analog scale) described at each stage but the maximum incidence in the incongruent mirror condition [*n* = 6 (15%)].

They used this finding to establish a cortical model of pain, in which the predicted sensory feedback (i.e., the efference copy) is compared with the actual sensory feedback. If the comparison results in a discrepancy, the mechanism induces pain or other sensory anomalies as a sign of distress, similar to the induction of nausea during a discordance of the visual and the vestibular sensory systems (Della-Morte and Rundek, 2012). Due to its clinical importance, the study by McCabe et al. (2005) aroused strong interest in the field of experimental pain research. In their comment published shortly afterward, Moseley and Gandevia (2005) challenged the far-reaching conclusions by McCabe et al. (2005) with reference to sample selection and potential induction of a response bias, although these criticisms were defended by McCabe et al. (2006). In another study, conflicting proprioceptive input has been shown to evoke several alterations in bodily sensation, but not pain (Moseley et al., 2006). However, this study explicitly excluded the visual domain, so it is unclear how these results relate to those reported by McCabe et al. (2005). Clinically, Daenen et al. (2010, 2012) used sensorimotor incongruence to evaluate the alterations in sensory integration in whiplash-associated disorders and regional pain syndrome and found an exacerbation of symptoms. Also, McCabe et al. (2007) reported increasing baseline pain and induced new sensory perceptions in patients suffering from fibromyalgia through the induction of a visuoproprioceptive conflict.

The study presented here aimed at providing additional clarification to the important initial findings of sensory changes in general and pain in particular, induced by a conflict between motor intention and visual feedback. We assessed quantitative in addition to qualitative data in a large sample of healthy participants and controlled response biases. Furthermore, we implemented additional conditions reducing the exertion of performing arm movements in order to evaluate the evoked sensory alterations without their potential blurring by physical fatigue. As we describe later, we specifically focused on an underreported phenomenon induced by sensorimotor incongruence: the alteration of perceived body integrity.

## Materials and Methods

### Participants

We investigated 113 subjects (74 female, 39 male, age range from 18 to 32, mean age 23.69, SD 2.92 years) with no current or past mental or physical illness and without any visible physical disfigurements, tattoos, or other markings on their hand and arms. All participants had normal or corrected-to-normal vision. The subject pool consisted mostly of University students from Mannheim, Heidelberg, and the surrounding region. All were informed about the movement tasks they would be expected to perform, but were held naïve with regard to the nature and purpose of the study and especially about the expected sensations during the mirrored movement task. All subjects were right-handed, as assessed by the Edinburgh Handedness Inventory (Oldfield, 1971). Self-reports about medical history, current medical conditions, and current medication or substance use, as well as any other conditions that might cause impaired visual, tactile, or proprioceptive processing (e.g., muscle fatigue from sports) were assessed using a standardized interview. Persons reporting any such conditions were excluded. Written informed consent was obtained prior to the study, which was approved by the Ethics Committee of the Medical Faculty Mannheim of the University of Heidelberg. The study conformed to the Code of Ethics of the World Medical Association (Declaration of Helsinki, sixth revision, 2008).

### Tasks

**Figure 1:**
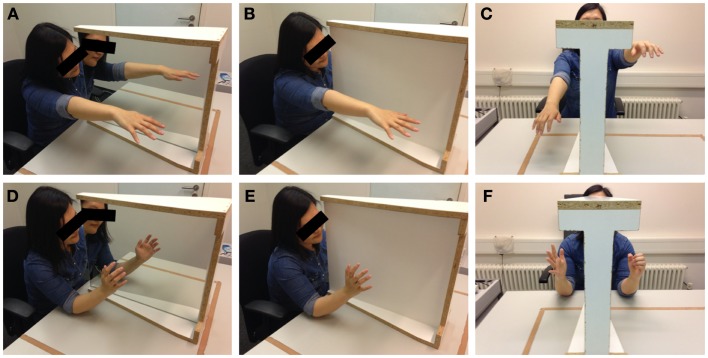
**Photographs of the experimental setup as used in the laboratory**. The mirror/whiteboard instrument is placed between the arms of the participant. The photographs depict the mirror **(A,D)** and whiteboard **(B,E)** conditions as well as the range of incongruent movements **(C,F)** for the arm **(A–C)** and hand **(D–F)** conditions. Photographs taken by author Robin Bekrater-Bodmann.

Before the experiment, participants were asked to remove any jewelry, watches, or asymmetric articles of clothing which might have helped them to identify a specific arm or hand as their left or right one. We used a specialized mirror construction with a large mirror surface (48 × 59 cm) on one side and a white, non-reflecting wooden control surface on the other side (see Figure 1). Its frame was symmetrical and allowed for the whole device to be turned around easily. While there was no condition without mirror or whiteboard (i.e., a condition with an unobstructed view of the opposing limb), and while the introduction of a wooden wall may already have an influence on body perception, the whiteboard condition eliminates any form of visual feedback of the intended or performed movement and is therefore considered a control condition for the purposes of this study. The situations in the experimental condition and the control condition were, apart from the presence of the mirror surface, identical. The participants were seated in such a way that their arms, if stretched horizontally, were in the middle between the top and the bottom of the mirror surface. It was always the dominant right arm that was hidden from view by the mirror construction, the non-dominant arm was always in full view. The reverse condition was not employed. The movement tasks were demonstrated and explained to the participants. One condition was a hand movement task identical to the movements commonly used in the mirror training for PLP (Ramachandran and Rogers-Ramachandran, 1996; Diers et al., 2010): here, the participant was asked to bend his or her fingers in a way that mimics the opening and closing of a fist, yet without letting the fingers touch each other or the palm of the hand to avoid additional tactile sensations. While doing this, the participant’s elbows were resting on the table and the hand was held in the middle between the top and bottom of the mirror surface. The movement was repeated for 20 s, at a frequency of 40 movements per minute, as guided by the sound of a metronome (13 movements). The other condition consisted of an arm movement similar to the one used by McCabe et al. (2005): the participants were asked to hold out their arms horizontally, palms downwards, and move them upwards and downwards, while taking care not to touch mirror or table. The duration and frequency of the movement were the same as in the hand condition. Regarding the initial arm position (elbow on the table for the hand condition, stretched arms for the arm condition), participants were allowed to adjust their arms in order to be as comfortable as possible, but were asked to keep in line with the standard positioning when the experimenters determined the adjustments to be too deviant.

After 20 s of movement, a verbal command was given to the participants: in the congruent condition, participants were asked to take a short break from the movement (approximately 1 s) and then continue the movement for 20 more seconds. In the arm condition, this break meant a return of the arms to a horizontal position before continuing the movement. In the hand condition, the break consisted of a relaxing of the fingers before continuing. This way, in the congruent condition, the participants performed congruent arm or hand movements for a total duration of 40 s.

The incongruent condition began the same way as the congruent condition, i.e., with 20 s of congruent movement. After this initial phase, participants were given a command to commence incongruent movements: in the arm condition, whenever one arm was held high, the other one was to be held low. In the hand condition, incongruence meant that one fist should be open while the other is closed. These motions, although they may be confusing at first, still needed to be performed fluidly. In order to ensure this, participants were informed about and demonstrated the required movements, and were told before each trial which command they had to expect. The sequence of these eight trials altogether (arms or hands, either in a congruent or incongruent manner, either in front of mirror or whiteboard) was randomized and all eight conditions were run once each for each participant. In all conditions and all phases, participants were instructed to direct their gaze to a horizontal black line that had been drawn across the middle of the surface (both on the mirror and on the whiteboard).

### Interviews

As done earlier in McCabe et al. (2005), participants were asked two questions after each trial: “How did that feel?” and “Were you aware of any changes in either limb?”, always in this sequence. In the study reported here, these questions were followed by 14 additional questions which were chosen according to the responses found by McCabe et al. (2005). These additional questions asked about sensory sensations such as pain, changes in temperature or weight, changes in the number of perceived limbs, etc., and were presented in randomized order. All questions are described in Table 1. The participants were asked to rate the intensity of those perceptions on a scale from 0 (not at all) to 10 (very strong). For each sensation, the participant had further to state in which body part he or she felt the sensation. According to McCabe et al. (2005) who found that the majority of induced sensations relate to the hidden limb, only somatosensory sensations attributed to the hidden right limb were included in further analyses.

**Table 1:** **Interview questions**.

Pain	Did you perceive any slight pain in either arm/hand during the experiment?
Itch	Did you perceive tickling or pins and needles in either arm/hand during the experiment?
Warmth	Did you feel your arm/hand getting warmer during the experiment?
Coldness	Did you feel your arm/hand getting colder during the experiment?
Lightness	Did you feel your arm/hand getting lighter during the experiment?
Heaviness	Did you feel your arm/hand getting heavier during the experiment?
Lost limb	Did you have the feeling of having less than two arms/hands during the experiment?
Extra limb	Did you have the feeling of having more than two arms/hands during the experiment?
Peculiarity	Did you perceive strange, not clearly identifiable sensations in either arm/hand during the experiment?
Pressure	Did you feel a change in pressure in either arm/hand during the experiment?
Shape	Did you perceive a change in length, circumference, or shape of either arm/hand during the experiment?
Numbness	Did you perceive numbness in either arm/hand during the experiment?
Nausea	Did you perceive nausea or dizziness during the experiment?
Other part	Did you perceive sensations in any other part of your body?

*After each run, these questions were asked, in randomized order, with the reference to arm or hand changed according to the condition. Question names refer to the naming system used in the text and figure descriptions*.

### Statistical analysis

An omnibus χ^2^ test of homogeneity for histograms was used to test for differences between the arm and hand conditions in the frequency of responses to the 14 items, regardless of intensity. The intensities of responses were compared across conditions for each item separately using Friedman’s two-way analyses of variance; results are reported with a level of *p* < 0.05. Further *post hoc* analyses were only conducted for items which exhibited a significant difference between experimental and control conditions using Wilcoxon signed rank tests. When necessary, we applied Bonferroni correction for multiple comparisons and all given *p*-values have been adjusted accordingly.

## Results

### Frequency of responses

**Figure 2:**
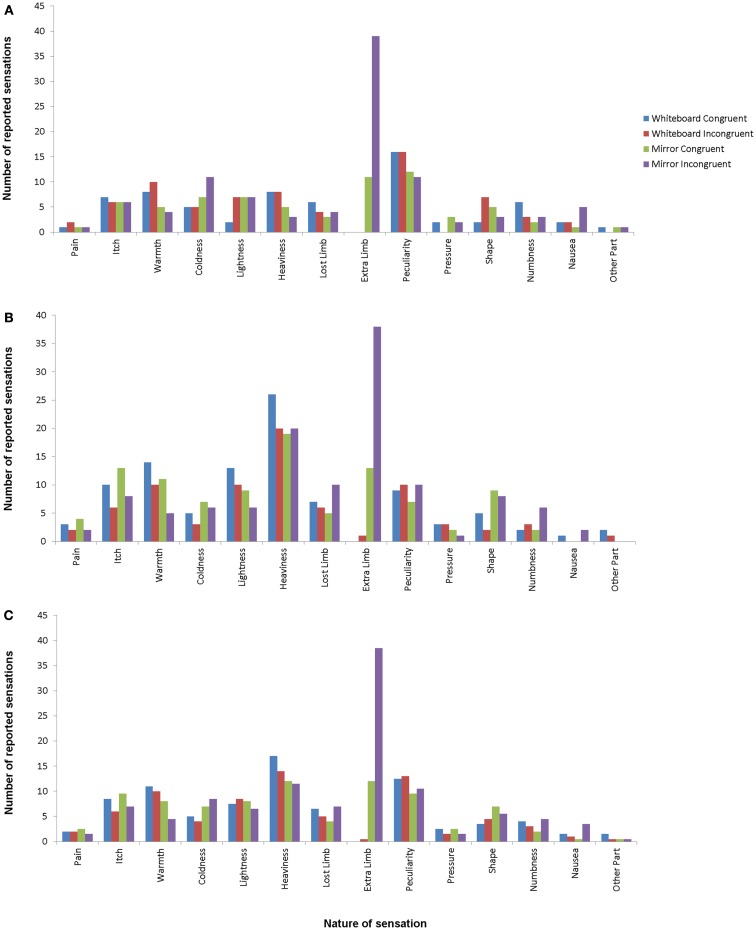
**Response frequencies for hand conditions (A), arm conditions (B), and average for all conditions (C)**. Different bars indicate different conditions, with mirror/incongruent being the experimental condition. Item definitions are given in Table 1.

Participants were asked to respond to each possible item. In the following section, the term “frequency” is used to indicate the number of all responses other than “No,” i.e., whenever the perception of the sensation was reported, regardless of its intensity. Figure 2 shows the frequencies of the responses for all sensations compared between the experimental and control condition. In the experimental condition (mirror/incongruent), 2 participants (1.8%) reported pain in the arm condition and 1 (0.9%) in the hand condition. The feeling of additional limbs was reported by 38 (33.6%) participants in the experimental arm condition and 39 (34.5%) participants in the experimental hand condition.

The omnibus χ^2^ tests did not reveal any significant differences between response frequencies in the arm and hand conditions (whiteboard incongruent: χ^2^_13_ = 17.44, *p* = 0.72; mirror incongruent: χ^2^_13_ = 21.40, *p* = 0.28; whiteboard congruent: χ^2^_13_ = 20.86, *p* = 0.32; mirror congruent: χ^2^_13_ = 14.87, *p* = 1.00). For this reason, we combined the arm and the hand conditions when looking for differences in sensation frequency between conditions.

A comparison of the conditions in this manner revealed a significant difference between the congruent and incongruent mirror condition (incongruent > congruent; χ^2^_13_ = 34.38, *p* < 0.05), but not between the congruent and incongruent whiteboard condition (χ^2^_13_ = 4.40, *p* = 1.00). Further comparisons revealed significant differences between the congruent mirror and congruent whiteboard condition (mirror > whiteboard; χ^2^_13_ = 35.20, *p* < 0.05) as well as the incongruent mirror and incongruent whiteboard condition (incongruent > congruent; χ^2^_13_ = 75.40, *p* < 0.05).

### Intensity of responses

**Figure 3:**
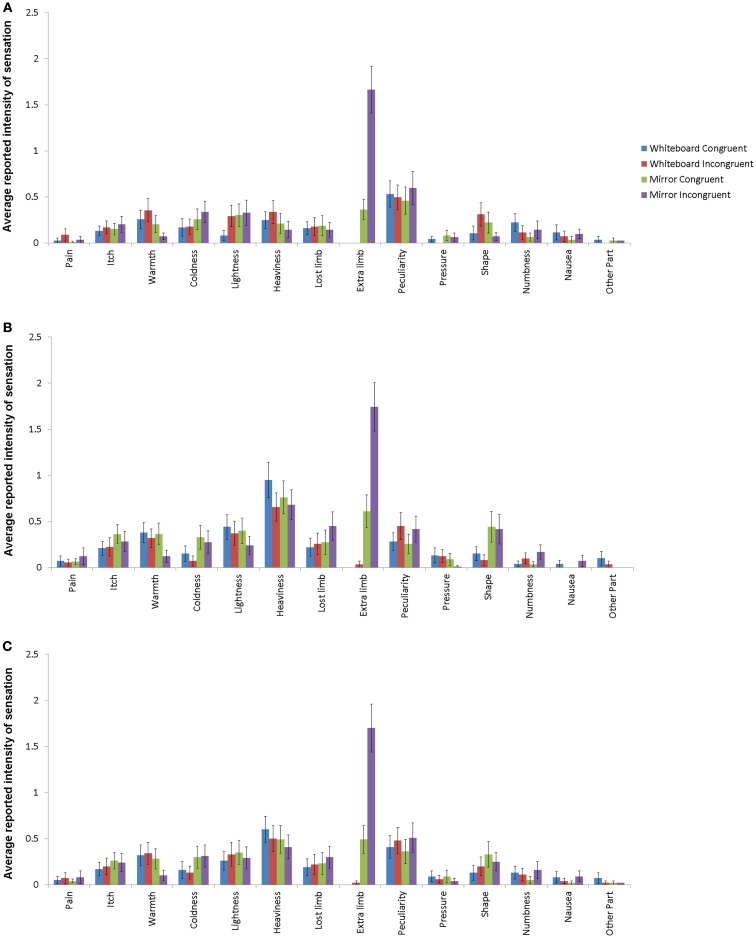
**Response intensities for hand conditions (A), arm conditions (B), and average for all conditions (C) with standard error depicted**. Different bars indicate different conditions, with mirror/incongruent being the experimental condition. Item definitions are given in Table 1.

Whenever a sensation was reported, participants were asked to determine its intensity, the values of which are analyzed in the following section. Due to the similarities of induced sensory alterations in the arm and hand conditions as indicated by the omnibus χ^2^ tests mentioned above, we used the arithmetic mean of intensities derived from the combination of both conditions. The intensity of the reported sensations varied widely depending on the nature of the perception. Figure 3 shows the reported intensities for all 14 sensations. By far the largest difference was visible for the feeling of supernumerary limbs: both in the arm and hand condition, this sensation showed the largest difference between the experimental and control conditions as well as the highest mean value of all responses in the experimental condition.

The comparison of the mean intensities of the 14 items across all conditions revealed a significant difference in only one of the items, which was the sensation of having an additional limb. For this item, the intensity was significantly higher in the experimental compared to all other conditions (χ^2^_3_ = 129.56, *p* < 0.001; mirror incongruent: *M* = 1.70, SD = 2.39; mirror congruent: *M* = 0.49, SD = 1.30; whiteboard incongruent: *M* = 0.02, SD = 0.19; whiteboard congruent: *M* = 0.00, SD = 0.00).

## Discussion

The goal of this study was to investigate as rigorously as possible the subtle reactions to an incongruent mirror movement experience. This was done by using a threefold approach in terms of standardization and control: (a) the specific effects of incongruent visual feedback were set against congruent movement conditions with and without visual feedback, (b) all movements of the participants were highly standardized, including the direction of the participants’ gaze, and (c) all responses were categorized to allow for statistical quantification. In addition, we used a large number of subjects in order to be able to document subtle or rare responses. This allowed us to gain several new insights into incongruent mirror feedback and body representation. The arm and hand conditions were not statistically different for response frequencies, suggesting that the magnitude of intentional body movements (hand movements are more subtle than arm movements) does not have an effect on the induction of illusory somatosensation. For the separate influences of the presence of a mirror or the presence of incongruence, we found that there was no significant difference in unusual somatosensory sensations across conditions, regardless of whether the condition only included incongruence or only included a reflected image. This suggests that sensorimotor conflict with a visual contribution does not induce more somatosensory perceptions than a non-conflicting condition, and that most of the reported experiences in this setup can be explained by an unfamiliar movement task, regardless of sensory feedback.

Pain was reported in less than 2% of participants, and was not more frequent during incongruent mirror feedback when compared to control conditions. The finding that pain was among the rarest sensations that were found in the study described here may suggest that the reported painful sensations might not have been genuine perceptions of pain, but rather unusual, surprising sensations (such as tingling or pins and needles) bordering on painfulness, which were interpreted or categorized as painful only by a small percentage of participants. Those unusual sensations that are equally spread over control and experimental conditions might be explained by the high degree of attention on the limbs elicited by the study setup, combined with an unusual movement task which might cause pain in some cases by straining muscles which are not commonly used for comparable tasks or durations. However, even if these were genuine painful sensations, they were not more frequent in the incongruent mirror condition. It is important to mention that a study by McCabe et al. (2007) has reported unusual sensations during mirror and whiteboard conditions, but not when the other limb was observed (i.e., unobstructed visual feedback, without any alteration or impairment of the participant’s visual field). From this perspective, both the mirror and the whiteboard conditions would be considered experimental, or interventional, conditions. However, our experimental setup demonstrates that the congruence or incongruence of the observed movements is not an influential factor on the frequency or intensity of reported pain. For some sensations, participants seem to have difficulties in determining whether the feeling is just uncomfortable or genuinely painful. Our questions to the participants were designed to provide the highest possible differentiation by including clear categorizations as well as assessing the intensity of the sensations. This mode of assessment and categorization of sensations that are odd and uncomfortable rather than painful, as well as the differences in establishing experimental and control conditions referenced above, may account for different findings in studies using comparable experimental setups (cf. McCabe et al., 2005; Moseley and Gandevia, 2005; Moseley et al., 2006).

The findings reported in the present study complement previous evidence of induced somatosensory perceptions elicited by sensory conflicts in so far as they suggest that a “pure” somatosensory incongruence (Moseley et al., 2006) as well as a cross-modal conflict with a visual contribution (the present study) does not appear to be sufficient to trigger substantial pain experiences in healthy volunteers. Nevertheless, this kind of incongruence might be able to affect the perceived integrity of one’s body. McCabe et al. (2005) already found that inducing a motor-sensory conflict leads to the sensation of owning more or less than two limbs, and participants stated that they had additional limbs only in the mirror/incongruent condition. The feeling of supernumerary limbs, in both the arm and hand condition, was by far the most frequently reported unusual sensation in the present study. The unique position of this sensation among the other responses is supported by evidence from other studies researching the reaction to unusual sensory feedback: in the experimental paradigm known as the rubber hand illusion (Botvinick and Cohen, 1998), one of the participant’s hands is hidden from view and replaced by a rubber hand. When this rubber hand is stimulated with a cotton swab while the hidden real hand receives tactile stimulation in a congruent manner, most participants report a feeling of ownership for the rubber hand. It is thought that, in this scenario, the incongruence between visual, tactile, and proprioceptive input is best resolved by the inclusion of the rubber hand into the body representation and that this external object basically replaces the actual hand in terms of body ownership (Longo et al., 2008). However, if the participant is allowed to observe his or her own stimulated hand *together* with the stimulated rubber hand, then participants no longer report the rubber hand as a replacement of their own hand, but instead perceive ownership for both the rubber hand and the actual hand (Guterstam et al., 2011). Another experimental study exploring sensorimotor incongruence using an adapted version of the rubber hand illusion (synchronous and asynchronous finger tapping of the rubber hand rather than stroking) demonstrated reduced ownership of the participant’s limb when the participant viewed the illusion of asynchronous finger tapping. Furthermore, participants reported significantly higher levels of pain, discomfort, feelings of peculiarity, and the perception of having an extra limb when they viewed asynchronous movements versus synchronous ones (Derbyshire et al., 2010). Interestingly, this kind of illusion is accompanied by shifts in topography of the primary somatosensory cortex, indicating that the subjective illusory sensation is related to changes on a neuronal level (Schaefer et al., 2009) in an area that, among other things, is responsible for multisensory integration (Schaefer et al., 2006). Our results suggest that not only a visuotactile conflict might alter the representation of the body, but that a sensorimotor conflict is also able to affect perceived body integrity. In order to investigate the phenomenon of supernumerary illusory limbs further, Folegatti et al. (2012) induced the rubber hand illusions with two rubber hands at the same time and found that only the one nearest to the body will be integrated into the participant’s body representation. It is interesting to note here that our mirror experiment is able to produce an illusory image (the mirrored hand) at the exact same location as the actual limb, which is something that the rubber hand illusion as described above is not able to do. This means that in the rubber hand illusion, there is a necessary contradiction between the location of the rubber hand and the participant’s proprioception. The central role of proprioception for the integration of body signals has been described by Vallar and Ronchi (2009), who discuss the sense of position as the defining factor for the occurrence of somatoparaphrenia, or delusional beliefs about parts of the body.

A distinction between body representations relating to perception (body image) and action (body schema) has been proposed earlier (Kammers et al., 2006). By this definition, our experiment is distinct from the rubber hand procedures cited above in that it aims at manipulating the action aspect of a limb (movement) instead of the perception aspect (by tactile stimulation). This means that in our study, body schema, rather than body image, is influenced by the experiment. A study by Newport et al. (2010) has demonstrated that a moving fake hand (which is comparable to observing a hand moving in a mirror) can be incorporated into both body image and body schema.

As described above, all our mirror movement setups began with congruent movements. Applying the results from the rubber hand paradigm, we assume that the mirrored arm or hand image replaces the actual arm or hand in the body representation during congruent movements, much like it is shown in the rubber hand setup. Minor incongruence (e.g., caused by imperfect two-hand coordination or shifts in balance) may lead to occasional sensations of a third limb, but it would be expected that most participants accept the mirror image as a replacement of their actual limb, because the “virtual” limb reacts completely congruently. However, as soon as the incongruent condition begins, a replacement is no longer sufficient to explain the mismatch in regard to sensory input and the body representation has to adjust to this novel situation. The results of this study show that, at least in some participants, the adjustment consists of accepting the visual reflection as a third limb. This means that the illusion itself is not being eliminated by the switch from congruent to incongruent movements: in both conditions, the mirrored hand is included into the body representation. The mode of integration, however, is changed according to the condition, and can alternate between a replacement of the hidden hand and the addition of a third hand.

This idea assumes that the sensation of additional limbs is specific to the situation of sensorimotor incongruence, which is consistent with the results of this study: in all of the whiteboard conditions, only one of the participants reported this feeling, compared to numerous reports of this sensation in the mirror conditions. Also, the frequency of this sensation, both in the arm and the hand conditions, is lowest in the whiteboard conditions, followed by moderate numbers in the congruent mirror condition and finally being named frequently in the incongruent mirror condition, as would be expected based on the theoretical background described above.

The fact that certain participants react with adding a third limb to their body representation and others do not, might have interesting implications for phantom sensations and PLP as well as the use of prosthetic limbs: in both cases, there is a large inter-individual variability that has not yet been completely explained. In the mirror treatment for PLP (Ramachandran and Rogers-Ramachandran, 1996), congruent mirror feedback is used to alleviate phantom pain. This method has been shown to reduce pain after several weeks of application (Chan et al., 2007). However, this and similar treatments do not seem to improve every patient’s condition (Weeks et al., 2010) and, if they work, the improvement is not always to the same degree (MacIver et al., 2008). A patient’s individual susceptibility to be influenced by visual feedback appears to be important in determining the efficacy of mirror therapy, with those who have the strongest immersion in the illusion gaining the greater analgesic benefit (Mercier and Sirigu, 2009). It is also known that a majority of prostheses are not used regularly, because patients reject them for reasons that are not entirely clear (Biddiss and Chau, 2007). These two phenomena might be linked on a conceptual level to the findings described here, where discordance between visual and somatosensory feedback could be integrated by some, but not all, participants. Since the ability to integrate a foreign object into the body representation is critical both in mirror therapy and in prosthesis use, its inter-individual variation may support or obstruct therapeutic efforts and thus demands further investigation. The notion of such an inter-individual variation is supported by the recent finding that the reaction to the rubber hand paradigm is stable over time, both on a behavioral and on a neuronal level (Bekrater-Bodmann et al., 2012). In addition, the fact that incongruent feedback facilitates the interpretation of the mirror image as a third limb has direct consequences on the practical application of mirror therapy for chronic pain syndromes such as PLP. One possible mechanism of this kind of treatment is the integration of information from both the sensorimotor and the visual systems, which contributes to the perception of the phantom limb (Hunter et al., 2003) and activates the body representation in sensorimotor cortex (Diers et al., 2010). Dysfunctional alterations in this cortical area might be involved in PLP (Flor et al., 2006). Consequently, a visual image replacing the missing limb might be an important factor for the efficacy of mirror treatment (Foell et al., 2011). Our data suggest that an incongruence between a mirror image and the phantom limb should lead to a rejection of the image as a replacement for the lost limb and may thus diminish the effects of the treatment. It is known that the size and shape of a phantom limb can deviate from that of a healthy limb (Giummarra et al., 2010) or be shortened by a so-called telescopic distortion (Cronholm, 1951; Ramachandran and Hirstein, 1998). If these effects cause the visual or proprioceptive differences between mirrored hand and phantom hand to become too large, the discrepancy may lead to the re-interpretation of the mirror image as a third limb rather than a replacement. Consequently, treatment effectiveness may be impaired, probably due to different representations in the sensory and motor cortices (cf. Schaefer et al., 2009; Diers et al., 2010). It would be interesting to investigate whether patients with a distinctive distortion of their phantom limb report less pain alleviation after the application of mirror therapy.

In conclusion, we did not find incongruent mirror feedback to elicit pain, which casts doubt on some current models for the origin of PLP and other chronic pain states. The sensation of additional limbs, however, is sensitive to the illusion created by this setup, and could prove to be highly important in the application of mirror feedback as a treatment for chronic pain.

## Conflict of Interest Statement

The authors declare that the research was conducted in the absence of any commercial or financial relationships that could be construed as a potential conflict of interest.
